# Identification of signature genes and relationship with immune cell infiltration in intervertebral disc degeneration

**DOI:** 10.3389/fgene.2025.1551124

**Published:** 2025-04-09

**Authors:** Kun Mu, JingChao Geng, Yu Dong, Wei Guo

**Affiliations:** ^1^ Department of Breast Surgery, Hebei Province Cangzhou Hospital of Integrated Traditional Chinese Medicine-Western Medicine, Cangzhou, China; ^2^ Department of Orthopaedics, Hebei Province Cangzhou Hospital of Integrated Traditional Chinese Medicine-Western Medicine, Cangzhou, China; ^3^ Department of Anaesthesiology, Hebei Province Cangzhou Hospital of Integrated Traditional Chinese Medicine-Western Medicine, Cangzhou, China; ^4^ Hebei Key Laboratory of Integrated Traditional and Western Medicine in Osteoarthrosis Research, Cangzhou, China; ^5^ Hebei Province Integrated Traditional Chinese and Western Medicine 3D Printing Technology Innovation Center, Cangzhou, China

**Keywords:** intervertebral disc degeneration, TOB1, immune cells infiltration, NLRP3 inflammasome, IL-1

## Abstract

**Background:**

Early diagnosis of intervertebral disc (IVD) degeneration is of great significance for prevention of the disease from progressing to a serious stage. This study aimed to investigate the signature genes and their association with immune cells in IVD degeneration.

**Methods:**

We analyzed differentially expressed genes (DEGs) in a dataset of IVD degeneration samples from the GEO database. Weighted gene coexpression network analysis (WGCNA) and DEGs were employed to pinpoint the key modules and IVD degeneration genes. Functional enrichment analysis was performed for these IVD degeneration genes. Signature genes were identified using least absolute shrinkage and selection operator (LASSO) analysis. Gene set enrichment analysis (GSEA) was used to explore signaling pathways related to signature genes, and CIBERSORT^®^ was used to classify immune cell infiltration. Function of the hub gene was confirmed by PCR, Western blotting and ELISA.

**Results:**

2,254 DEGs were identified from GSE56081, and WGCNA grouped the data into 9 modules. MEbrown module had a significant correlation with IVD degeneration (cor = 0.99, *P* = 8.00 × 10^−8^). LASSO analysis selected HSPA1B, TOB1, ECM1, PTTG1IP as signature genes with excellent diagnostic efficiency. Furthermore, we assessed the diagnostic efficacy of every signature gene in predicting IVD degeneration using an external validation group (GSE70362). The results showed that two of the signature genes (TOB1, ECM1) had significant diagnostic effect in predicting the degeneration of IVD. GSEA analysis showed TOB1 and ECM1 involve in NOD like receptor signaling pathway, phenylalanine metabolism. Ether lipid metabolism, glycosaminoglycan biosynthesis keratin sulfate, RNA degradation pathway. CIBERSORT^®^ suggested TOB1 and ECM1 may participate in immune cells infiltration. Finally, we identified TOB1 as a crucial molecule in the process of NP cell pyroptosis and NLRP3 inflammasome activation.

**Conclusion:**

TOB1 may show remarkable diagnostic performance in IVD degeneration and may be implicated in the infiltration of immune cells.

## Introduction

Low back pain (LBP) is one of the most common musculoskeletal disorders that negatively affects people of all ages and socioeconomic groups (Knezevic et al., 2021). LBP is the most common reason for medical consultation worldwide, and it is one of the leading causes of disability (Knezevic et al., 2021). Studies have shown that LBP can be caused by a number of reasons, however, intervertebral disc (IVD) degeneration is thought to be the most important of them (Martin et al., 2008; Livshits et al., 2011). IVD degeneration is a complex multifactorial condition with a high prevalence rate in the general population. According to epidemiological studies, the prevalence of IVD degeneration ranges from 20% to 80% worldwide, with a higher incidence rate in older adults (Cheung et al., 2009). Several risk factors have been identified to contribute to the development of IVD degeneration, including genetic factors, aging, mechanical loading, and environmental factors such as smoking and obesity (Adams and Roughley, 2006). Early diagnosis of IVD degeneration is of great significance for timely and targeted treatment, relief of pain, and prevention of the disease from progressing to a serious stage. However, there are currently no highly reliable signature genes available for the diagnosis of early IVD degeneration.

Currently, the cause and mechanisms of IVD degeneration remain unclear. However, apoptosis, imbalanced cell ratios between senescent and active cells, abnormal extracellular matrix (ECM) degradation, and inflammatory cascades in IVD cells are frequently cited as potential factors. Recent studies have focused on the role of the immune system in the development of IVD degeneration (Sun et al., 2020). The immune system plays a crucial role in maintaining tissue homeostasis, and immune dysregulation has been implicated during the development of degenerative changes in IVD. In particular, the inflammasome, a multiprotein complex that regulates the activation of inflammatory cytokines, has been shown to play a key role in the initiation and progression of IVD degeneration (Chao-Yang et al., 2021). Studies demonstrated that abnormal production of pro-inflammatory molecules by nucleus pulposus (NP) and annulus fibrosus (AF) cells, as well as macrophages, T cells, and neutrophils leads to the development of IVD degeneration (Yamamoto et al., 2013; Rand et al., 1997; Kepler et al., 2013). TNF-α was associated with disc herniation and nerve irritation and growth (Hayashi et al., 2008; Murata et al., 2006), both TNF-α and IL-1β cause increased expression of genes that encode matrix-degrading enzymes (Le Maitre et al., 2005; Wang et al., 2011; Le Maitre et al., 2007a). It has been demonstrated that the degenerated disc tissue shows an increase in the expression of IL-1β and IL-1R (Le Maitre et al., 2005; Le Maitre et al., 2007b). Some studies suggest that drugs that inhibit inflammasome activation or the release of inflammatory factors can attenuate the progression of IVD degeneration (Chao-Yang et al., 2021).

As the avascular organ at the center of the IVD, NP tissue is anatomically isolated from the host immune system, making it a privileged organ of immunity. The rupture of the AF disrupts the NP-blood barrier, leading NP exposing to the host and leads to immune cell infiltration and inflammatory response. Immune cell infiltration has been shown to contribute to the progression of IVD degeneration (Wang et al., 2021). These infiltrating immune cells, such as neutrophils and T cells (CD4^+^, CD8^+^), will release inflammatory cytokines, further worsening the inflammatory response cascade. Previous studies demonstrated that TOB1 is an anti-proliferative protein of Tob/BTG family and typically involved in the immune response and T cell activation (Yu et al., 2022; Lin et al., 2022); And ECM1 was highly expressed in tissue-infiltrated macrophages under inflammatory conditions and involved in the activation of nuclear factor κB signal pathway (Zhang et al., 2020; Lv et al., 2022). However, the role of TOB1 and ECM1 in modulating immune responses in IVD degeneration remains unclear. While immune and inflammation regulation therapy is still in a developing field, further investigation of immune-related inflammatory responses may provide insights for IVD degeneration treatment.

The objective of this research was to identify signature genes and potential therapeutic targets for the management of IVD degeneration through bioinformatic analysis. Transcriptome microarrays were utilized in this study to detect signature genes in individuals with IVD degeneration, which offers novel perspectives on the diagnosis and treatment of the disease. Moreover, we investigated the effects of TOB1 overexpression on human NP cells pyroptosis and NLRP3 inflammasome activation.

## Materials and methods

### Clinical specimens

Human lumbar degenerative NP specimens were obtained from 10 patients with IVD degeneration undergoing discectomy. The control samples were taken from 10 age- and sex-matched patients with fresh traumatic vertebral fracture undergoing decompressive surgery because of neurological deficits.

### Data sources

For this study, two datasets named GSE56081 (contained both mRNA and lncRNA data) and GSE70362 were downloaded from Gene Expression Omnibus (GEO). As a training set, the mRNA data of GSE56081 contained 5 NP samples with IVD degeneration and five normal samples that collected from degenerative lumbar and human normal (cadaveric donors) NP respectively (Lan et al., 2016). The validation group consisting of 16 NP samples with IVD degeneration and eight normal samples that obtained by McGill Scoliosis and Spine Group from human lumbar discs through organ donation program of Transplant Quebec in accordance with the local and institutional ethical guidelines were downloaded from GSE70362 (Kazezian et al., 2015). All of the aforementioned studies have obtained approval from the appropriate institutional review boards, and the participants have provided informed consent.

### Differentially expressed genes identification

The limma package in R was used to identify differentially expressed genes (DEGs) between IVD degeneration NP samples and normal NP samples from GSE56081 dataset with false discovery rate (FDR) *P* < 0.05 and |fold change (FC)| > 1 as threshold. These DEGs were displayed in the volcano plot and the top 50 upregulated and top 50 downregulated DEGs were demonstrated in the heatmap.

Weighted gene co-expression network analysis (WGCNA) was used to identify IVD degeneration genes. A co-expression network in GSE56081 dataset by weighted gene co-expression network analysis (WGCNA) was conducted in this study based on scale-free topological criteria (Langfelder and Horvath, 2008). The soft threshold power and adjacency relationship was calculated by the pickSoftThreshold function of WGCNA package. Then, the adjacency matrix is transformed into topological overlap matrix (TOM), and the corresponding differences is figured up to carry out hierarchical clustering analysis. Co-expressed gene modules were authenticate by dynamic tree cutting with the minimum module size of 60 (scale-free topology fit index >0.9 and cut height = 0.95). Then this study measured the association between gene modules and disc degeneration by gene significance values and module membership values, and ultimately identified key modules and IVD degeneration genes.

### IVD degenerative genes enrichment analysis

This study performed functional enrichment analysis based on Gene Ontology (GO) and (Kyoto Encyclopedia of Genes and Genomes) KEGG analysis using R package clusterProfiler. And the top 10 GO and top 10 KEGG signaling pathways were displayed.

### Single cell RNA-seq data analysis

Raw RNA-seq data (GSE165722) were processed according to bioinformatics analysis principles of the previous studies (Guo et al., 2023; Gao et al., 2022). Cells samples with count of fewer than 200 genes or more than 20% of mitochondrial genes were filtered out. t-distributed stochastic neighbor embedding (t-SNE) analysis (Cieslak et al., 2020), K-mean clustering and hierarchical clustering methods were applied to analyze the data.

### Signature gene identification

We identified candidate signature genes by the intersection of IVD degeneration genes and specifically expressed marker genes of degeneration NP cells. Then, this study used machine learning algorithms, called least absolute shrinkage and selection operator (LASSO) to screen signature genes. The screen out of genes were considered to be the signature genes of IVD degeneration. The diagnostic efficiency of these signature genes was evaluated by the area under curve (AUC) of the receiver operating characteristic curve (ROCs). An AUC greater than 0.8 indicates a good diagnostic effect.

### GSEA analysis

In order to determine the relationship between signature genes and signaling pathways, this study classified the IVD degeneration dataset in terms of the median expression of signature genes. Then the gene set enrichment analysis (GSEA) was performed on different subgroups with adjusted *P* < 0.05.

### Immune cell infiltration analysis

This study applied the linear support vector regression principle and CIBERSORT^®^ method to deconvolute the expression matrix of 22 subtypes of human immune cells, with the aim of examining the dissimilarities in immune cells between normal subjects and patients with IVD degeneration. Next, this study filtered for immune cells that had significant differences in infiltration between IVD degeneration patients and normal subjects, and then used Spearman correlation analysis to investigate their relationship with signature genes.

### Human NP cell culture

The NP tissue specimens were isolated and sectioned into 1 mm fragments, followed by digestion of the NP tissue using a solution of 0.25% pronase and 0.2% collagenase type II at a temperature of 37°C. The resulting digested suspension was then filtered through a mesh with a pore size of 70 μm and subsequently cultured in DMEM medium supplemented with 10% fetal bovine serum and 1% penicillin-streptomycin, maintained at a CO_2_ concentration of 5% and a temperature of 37°C.

### Cell counting kit-8 (CCK-8) assay

Cell proliferation was evaluated using the CCK-8. Human NP cells, which were transfected with TOB1, were plated into 96-well plates and incubated for durations of 12, 24, 36, 48, 72, and 96 h. Following this incubation, CCK-8 was introduced to the cells, and they were allowed to incubate for an additional 3 h. The absorbance was subsequently measured at a wavelength of 450 nm.

### ELISA

The contents of human IL-1β in the cell culture supernatant were detected according to the manufacturer’s instructions.

### Western blotting

Cells were subjected to lysis using a buffer that included a protease inhibitor, 0.25 M Tris-HCl, 20% glycerol, 4% sodium dodecyl sulfate (SDS), and 10% mercaptoethanol, adjusted to a pH of 6.8. Equal amounts of total protein (10 μg) were resolved on a 10%–12% SDS-polyacrylamide gel and subsequently transferred to a polyvinylidene fluoride membrane via electroblotting. Following this, blocking was conducted using a solution of 5% non-fat milk in Tris-buffered saline with 0.1% Tween-20 (TBST) at room temperature for 1 hour. This was succeeded by an overnight incubation with primary antibodies in TBST supplemented with 5% non-fat milk at 4°C. Afterward, a secondary antibody was applied at room temperature for 1 hour, and the Western blotting procedure was executed utilizing an enhanced chemiluminescence detection system.

### Quantitative reverse-transcription PCR (RTqPCR)

Following chloroform extraction and subsequent precipitation, DNA was eliminated from the samples using DNase I treatment. This was succeeded by the reverse transcription of the purified RNA, which was facilitated by RevertAid reverse transcriptase. Primers for reverse transcription, incorporating CMV promoter sequences, were specifically designed to target designated genes. RT-qPCR was conducted utilizing both the AriaMx Real-time PCR system and the QuantStudio Real-time PCR system. The primers employed in this investigation are provided in Supplementary Table S4.

### Cell transfection

Lasmids or siRNA were transfected into third-generation NP cells using Lipofectamine 3,000 (Invitrogen), in accordance with the manufacturer’s instructions and as previously described (Guo et al., 2020). Cells were harvested 48 h post-transfection.

### Statistical analysis

All statistical evaluations in the current investigation were conducted using R software (version 4.1.2). Unless specified otherwise, a *P* value of less than 0.05 was considered to be statistically significant, and all *P* values were two-tailed.

## Results

### Identification of DEGs between IVD degeneration and normal samples

**FIGURE 1:**
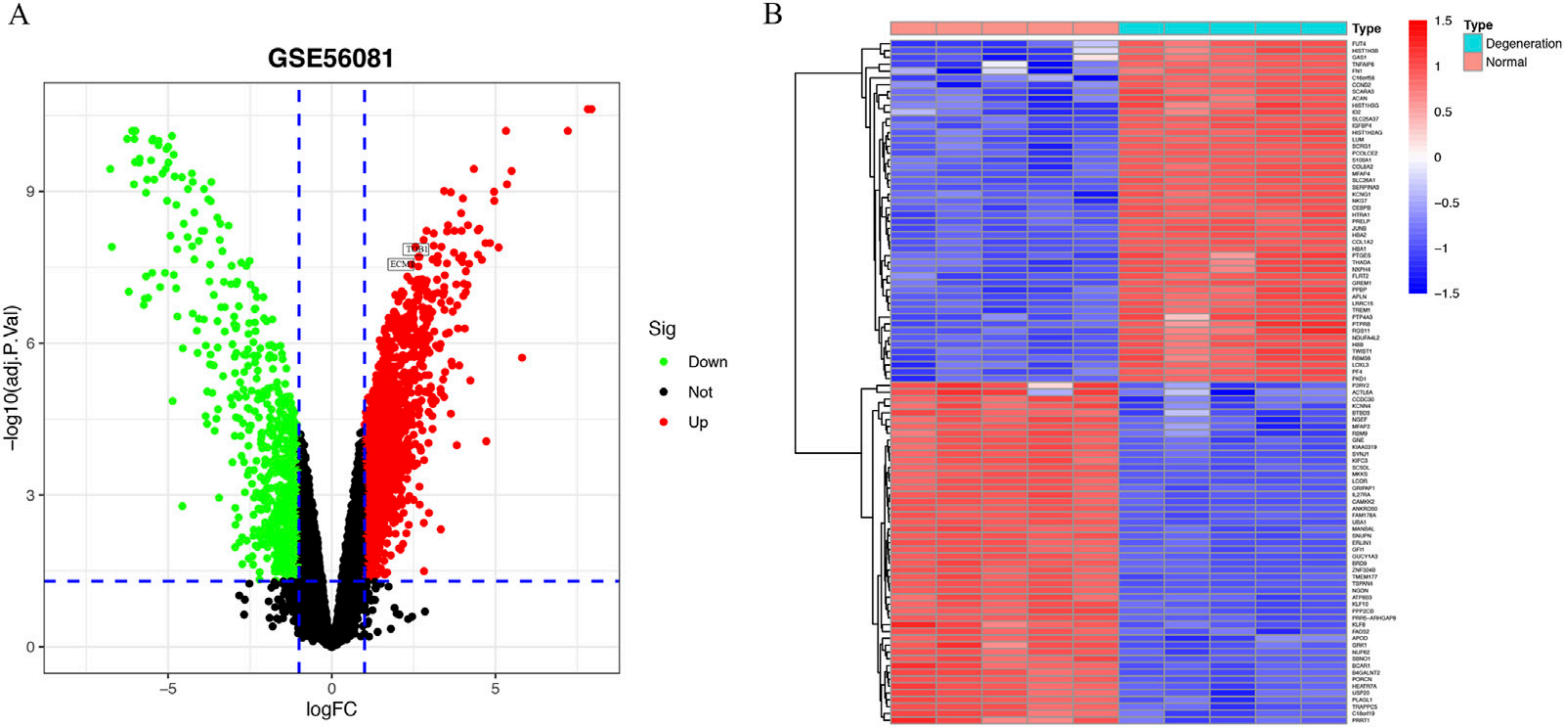
Identification of DEGs in IVD degeneration. **(A)** Volcano showed expression of DEGs between the IVD degeneration and normal NP samples. **(B)** The heatmap showed the top 50 upregulated differentially expressed genes and 50 downregulated DEGs.

A total of 2,254 DEGs were discovered in the GSE56081 dataset (Supplementary Table S1), which comprised of 1,411 upregulated and 843 downregulated genes with the FDR P value < 0.05 and |log2 (fold change, FC)| > 1 (Figure 1A). The heatmap demonstrated the leading 50 upregulated and 50 downregulated DEGs between IVD degeneration and normal samples (Figure 1B).

### Detection of genes associated with IVD degeneration via WGCNA

**FIGURE 2:**
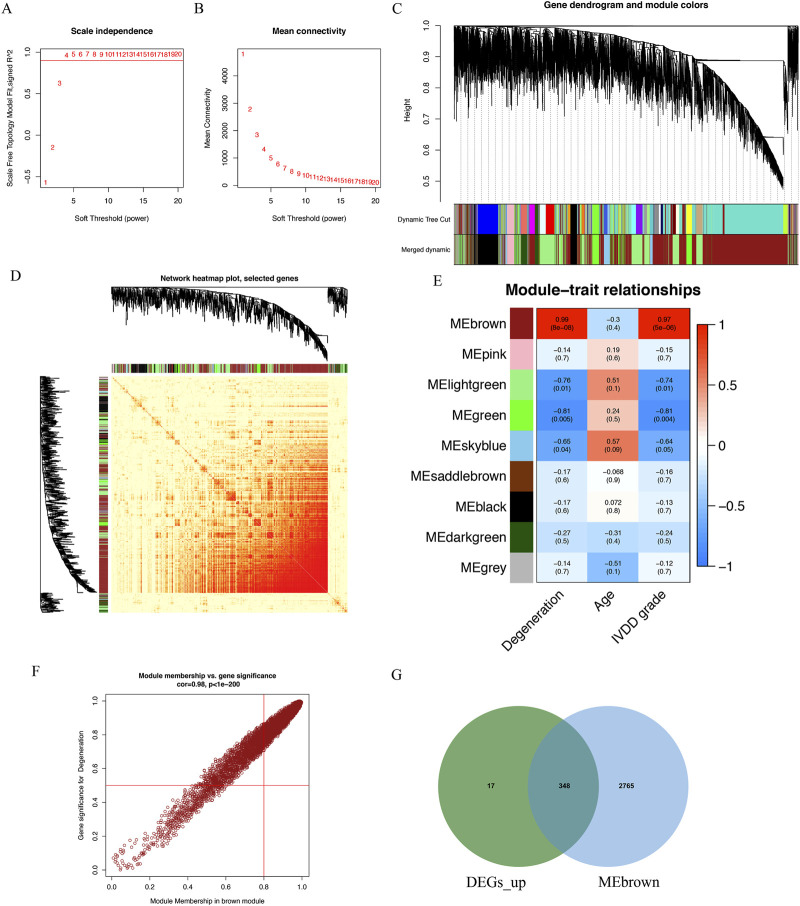
Identification of IVD degeneration genes by WGCNA. **(A)** The soft threshold power of WGCNA. **(B)** The mean connectivity of WGCNA. **(C)** The cluster dendrogram of WGCNA. **(D)** Network TOM heatmap plot of WGCNA. **(E)** The clustered modules of WGCNA. **(F)** A scatterplot of Gene Signifificance for weight vs. Module Membership in the brown module. **(G)** The venn plot showed the intersection between upregulated DEGs and genes in MEbrown module.

The WGCNA package in R software was employed to examine IVD degeneration and normal samples, and to establish a scale-free co-expression network. A soft threshold power of 15 was determined, with a scale-free index of 0.9 and a relatively favorable mean connectivity (Figures 2A,B). The cluster dendrogram was presented in Figure 2C. The heatmap of gene co-expression network is shown in Figure 2D. Ultimately, the data was classified into nine modules, and the correlation between each module and IVD degeneration was assessed. The findings revealed that the MEbrown module had a significant association with IVD degeneration (Figure 2E). The MEbrown module consisted of 5,590 genes (Supplementary Table S2), and was deemed as a critical module associated with IVD degeneration. It can be observed that the genes highly correlated with the brown module (top right corner) are also highly correlated with degeneration, while the genes with low module correlation (bottom left corner) are also not associated with degeneration (Figure 2F). Figure 2G displays the overlap between the upregulated differentially expressed genes with logFC > 2 and the genes located in the top right corner of Figure 2F. A total of 348 genes were identified, and these genes were regarded as IVD degeneration genes (Supplementary Table S3).

### Function enrichment analysis of IVD degeneration genes

**FIGURE 3:**
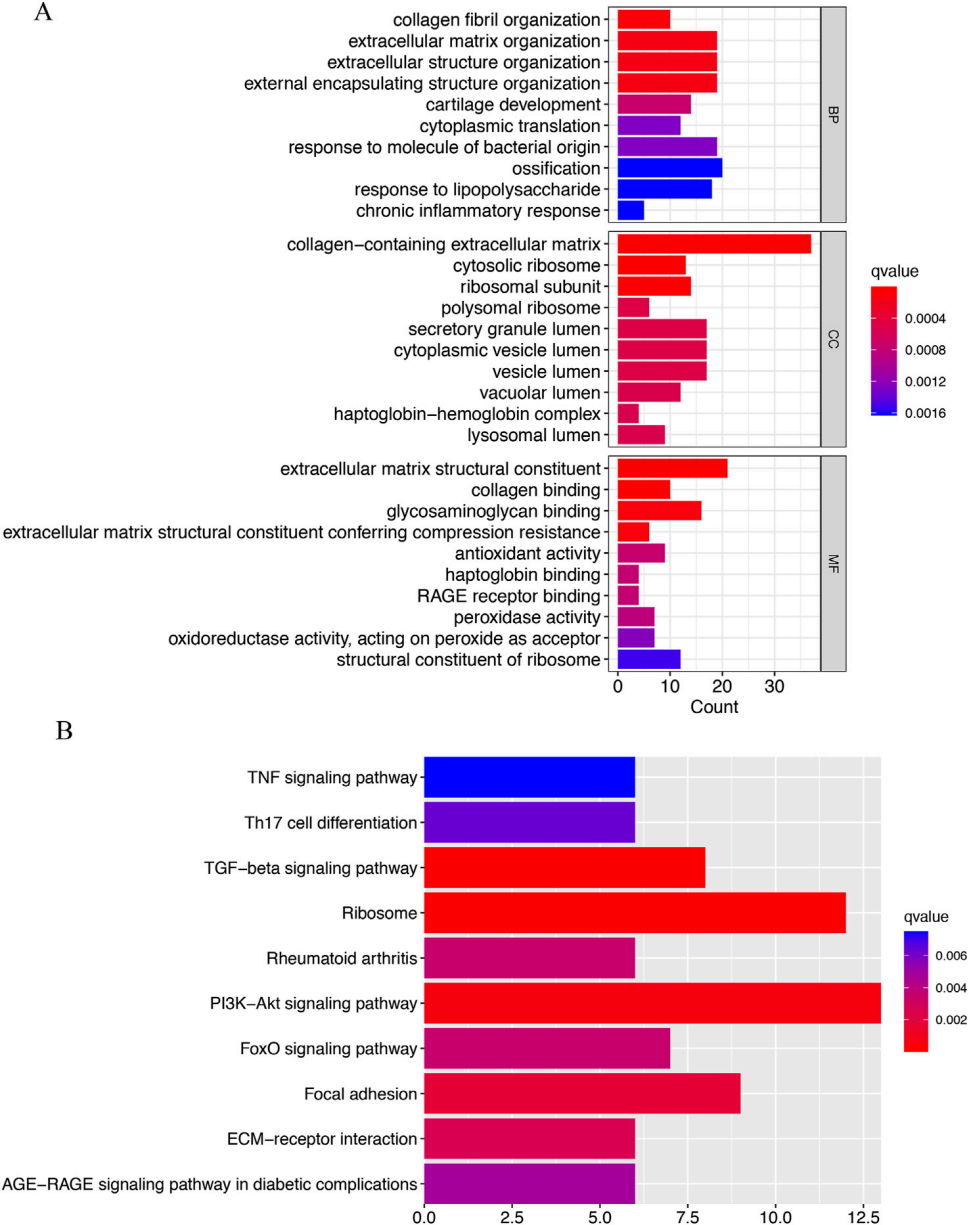
Functional enrichment analysis of IVD degeneration genes. **(A)** The top 10 functional enrichment in BP, CC, and MF analysis, respectively. **(B)** The KEGG analysis of IVD degeneration genes.

The GO analysis was categorized into three groups: BP, CC, and MF. The BP analysis revealed that collagen fibril organization, extracellular matrix organization, extracellular structure organization, external encapsulating structure organization, cartilage development, cytoplasmic translation, response to molecule of bacterial origin, ossification, response to lipopolysaccharide, chronic inflammatory response were significantly enriched; In the CC analysis, collagen-containing extracellular matrix, cytosolic ribosome, ribosomal subunit ranked among the top three positions; Furthermore, extracellular matrix structural constituent, collagen binding, glycosaminoglycan binding, extracellular matrix structural constituent conferring compression resistance played a crucial role in MF (Figure 3A). According to the KEGG analysis, the top three enriched pathways were (the phosphatidylinositol 3′-kinase) PI3K-Akt signaling pathway, Ribosome, and TGF-beta signaling pathway (Figure 3B).

### Single-cell transcriptomic profiles of the NP tissue from IVD degeneration and control samples

**FIGURE 4:**
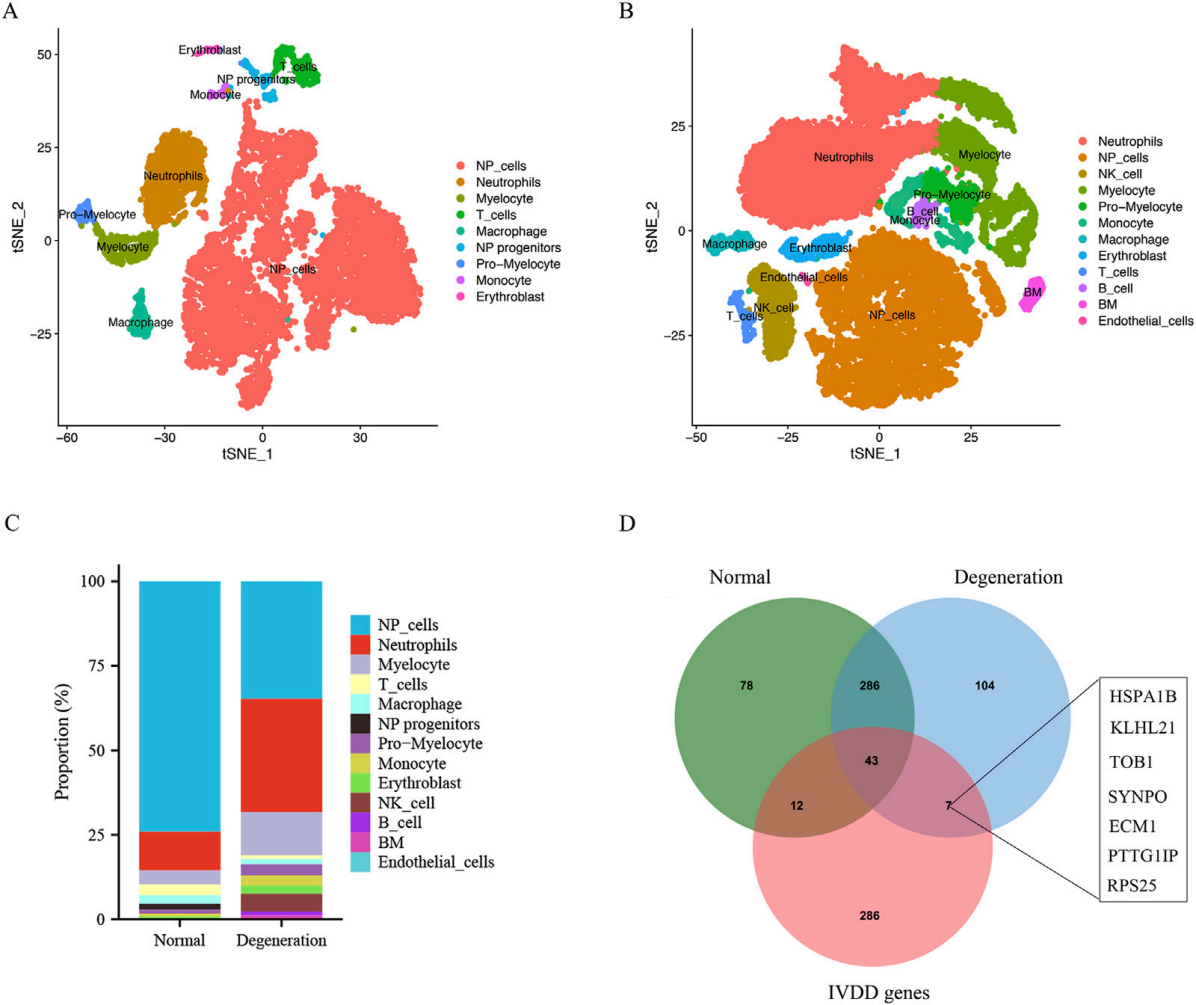
Single cell sequencing data analysis and identification of different cell types. **(A and B)** t-SNE projections and cell type annotation of NP tissue. **(C)** Bar plot showing the percentage of each cell type in IVD degeneration and normal NP samples. **(D)** The venn plot showed the intersection of normal NP cell marker genes, degenerative NP cell markers and IVD degeneration genes.

This study utilized (Single-cell RNA sequencing) scRNA-seq data (GSE165722) to analyze and identify different cellular subpopulations in normal and degenerated IVD NP tissue, and presented the outcomes using a dimensionality reduction approach (t-SNE). In normal NP tissue, this study identified nine different cellular subtypes (Figure 4A), whereas in degenerated NP tissue, this study determined a total of 12 distinct cellular subtypes (Figure 4B); these subtypes were determined via singleR and cell markers. Next, this study further quantified the relative proportions of various cellular types in normal and degenerated NP tissue samples. Figure 4C shows that in degenerated NP tissue, the proportion of NP cells is significantly reduced, while the proportion of various immune cells is significantly increased, especially neutrophils. Through analysis of the intersection of normal NP cell marker genes, IVD degeneration genes, and degenerated NP cell marker genes, this study found that seven signature genes belonged to both degenerated NP cell marker genes and IVD degeneration genes, but not to normal NP cell marker genes. This suggests that these signature genes may play a role in the process of IVD degeneration and may be potential targets for further research in understanding the biology and function of NP cells (Figure 4D).

### Selection of signature genes through LASSO analysis

**FIGURE 5:**
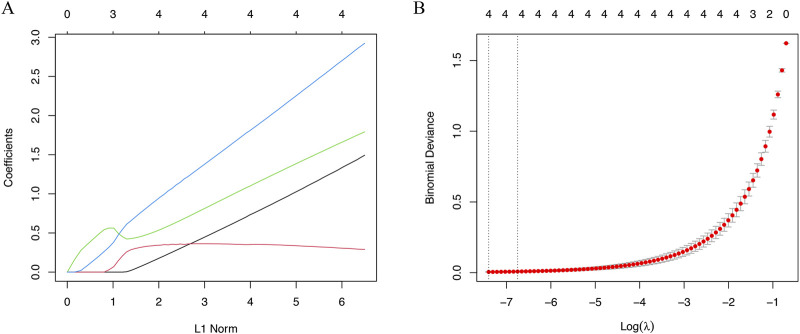
The machine algorithms for signature genes. **(A)** Penalty plot of the LASSO model with error bars denoting standard errors. **(B)** LASSO plot showed the variations in the size of coefficients for parameters shrank as the value of k penalty increased.

In this study, LASSO analysis was applied to screen out signature genes from signature genes of IVD degeneration. And a total of 4 signature genes were identified, including HSPA1B, TOB1, ECM1, PTTG1IP (Figures 5A,B).

### Diagnostic efficacy of signature genes in predicting IVD degeneration

**FIGURE 6:**
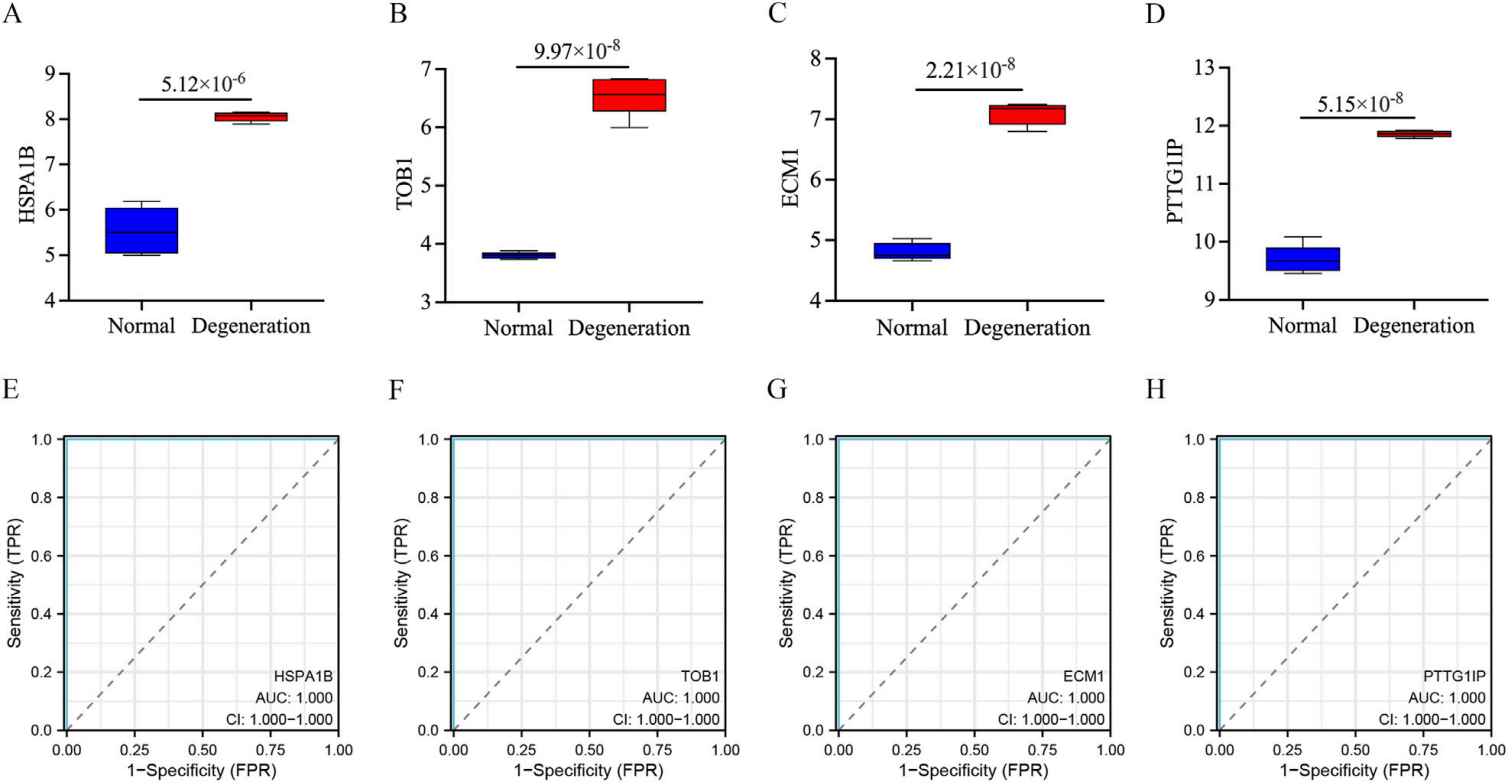
The performance of the signature genes in GSE56081. **(A–D)** The expression of signature genes between the IVD degeneration and normal NP samples. **(E–H)** ROC showed the diagnostic performance of the signature genes.

**FIGURE 7:**
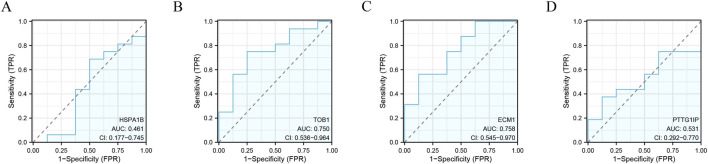
The performance of the signature genes in GSE70362. **(A–D)** ROC showed the diagnostic performance of the signature genes.

The identified signature genes exhibited elevated expression levels in IVD degenerated NP tissue compared to normal samples, indicating that these genes might have a potential function in pediatric IVD degeneration (Figures 6A–D). Figures 6E–H shows the area under curve (AUC) of the signature genes. Furthermore, this study assessed the diagnostic efficacy of every signature gene in predicting IVD degeneration using an external validation group (GSE70362). The results showed that two of the signature genes (TOB1, ECM1) had significant diagnostic effect in predicting the degeneration of IVD (Figures 7A–D). These observations suggested that TOB1 and ECM1 possessed remarkable diagnostic effectiveness in predicting IVD degeneration.

#### Gene set enrichment analysis

**FIGURE 8:**
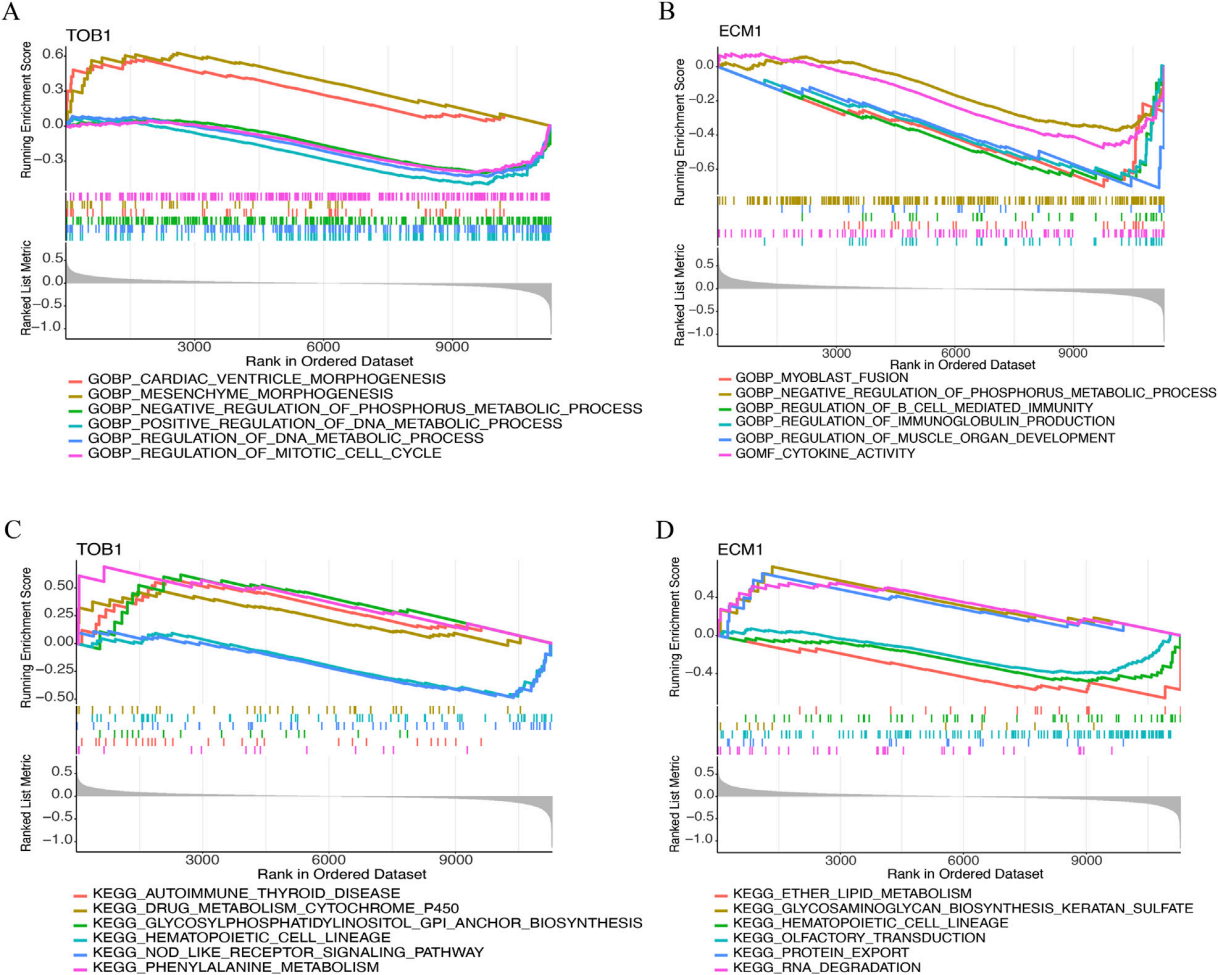
The GSEA of TOB1 and ECM1 in IVD degeneration. **(A and B)** GO enrichment results of TOB1 and ECM1. **(C and D)** KEGG enrichment results of TOB1 and ECM1.

This study conducted a GSEA analysis to examine the GO terms and signaling pathways associated with the TOB1 and ECM1. The top six GO terms were exhibited in Figures 8A,B. The results showed that TOB1 was markedly associated with cardiac ventricle morphogenesis (*P* = 1.71 × 10^−4^, NES = 1.89), mesenchyme morphogenesis (*P* = 2.01 × 10^−4^, NES = 1.90), negative regulation of phosphorus metabolic process (*P* = 1.36 × 10^−4^, NES = −1.58), regulation of DNA metabolic process, regulation of mitotic cell cycle (*P* = 2.40 × 10^−5^, NES = −1.84) (Figure 8A). The expression of ECM1 was markedly related to myoblast fusion (*P* = 8.82 × 10^−5^, NES = −2.02), negative regulation of phosphorus metabolic process (*P* = 1.56 × 10^−4^, NES = −1.60), regulation of B cell mediated immunity (*P* = 9.96 × 10^−5^, NES = −2.01), regulation of immunoglobulin production (P = 6.16 × 10^−6^, NES = −2.09), regulation of muscle organ development (*P* = 1.55 × 10^−4^, NES = −2.00), cytokine activity (*P* = 5.10 × 10^−5^, NES = −1.86) (Figure 8B). Figures 8C,D showed the top six signal pathways. The results indicated that TOB1 was significantly correlated with autoimmune thyroid disease (*P* = 6.61 × 10^−3^, NES = 1.66), drug metabolism cytochrome P450 (*P* = 0.02, NES = 1.47), glycosylphosphatidylinositol GPI anchor biosynthesis (*P* = 0.01, NES = 1.60), hematopoietic cell lineage (*P* = 0.02, NES = −1.51), NOD like receptor signaling pathway and phenylalanine metabolism (*P* = 0.02, NES = −1.48) (Figure 8C). The expression of ECM1 was markedly related to ether lipid metabolism (*P* = 6.92 × 10^−3^, NES = −1.68), glycosaminoglycan biosynthesis keratin sulfate (*P* = 5.67 × 10^−3^, NES = 1.79), hematopoietic cell lineage (*P* = 6.50 × 10^−3^, NES = −1.63), olfactory transduction (*P* = 4.89 × 10^−3^, NES = −1.55), protein export (*P* = 4.36 × 10^−3^, NES = 1.75), RNA degradation (*P* = 3.25 × 10^−3^, NES = 1.75) (Figure 8D).

### Immune cell infiltration

**FIGURE 9:**
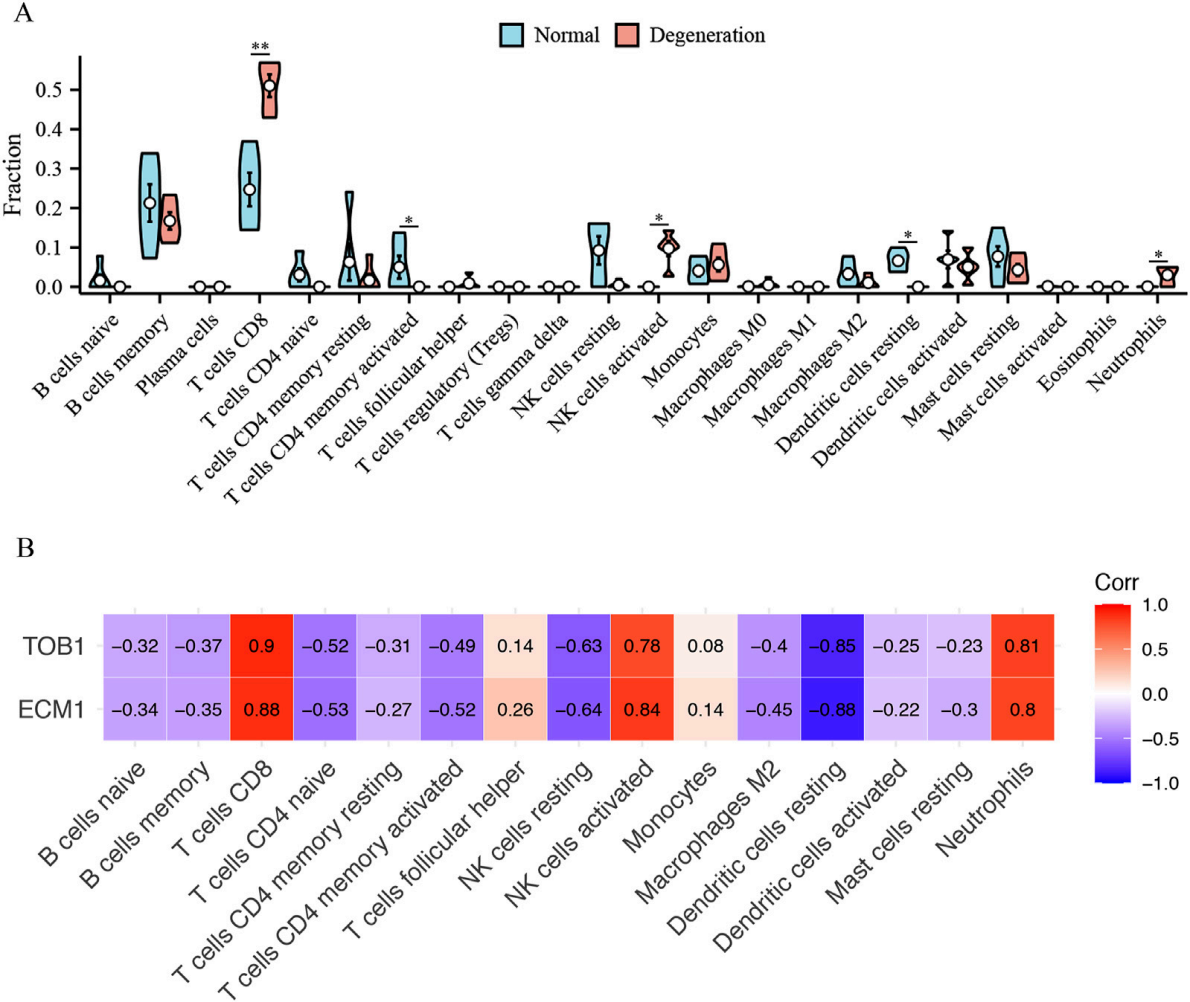
The immune cell infiltration association with TOB1 and ECM1. **(A)** The immune cell infiltration between the IVD degeneration and normal NP samples. **(B)** The association between signature genes (TOB1 and ECM1) and significantly different immune cell infiltration. *P < 0.05, **P < 0.01.

Immunological characteristics were assessed based on the infiltration of immune cells. In contrast to healthy individuals, those with IVD degeneration exhibit elevated levels of T cells CD8, (natural killer) NK cells activated, neutrophils, and reduced T cells CD4 memory activated, dendritic cells resting (Figure 9A). Notably, both TOB1 and ECM1 were positively correlated with the infiltration of CD8^+^ T cells, NK cells, neutrophils, and negatively correlated with the infiltration of CD4^+^ T cells and dendritic cells (Figure 9B).

### TOB1 overexpression activated NLRP3 inflammasome

**FIGURE 10:**
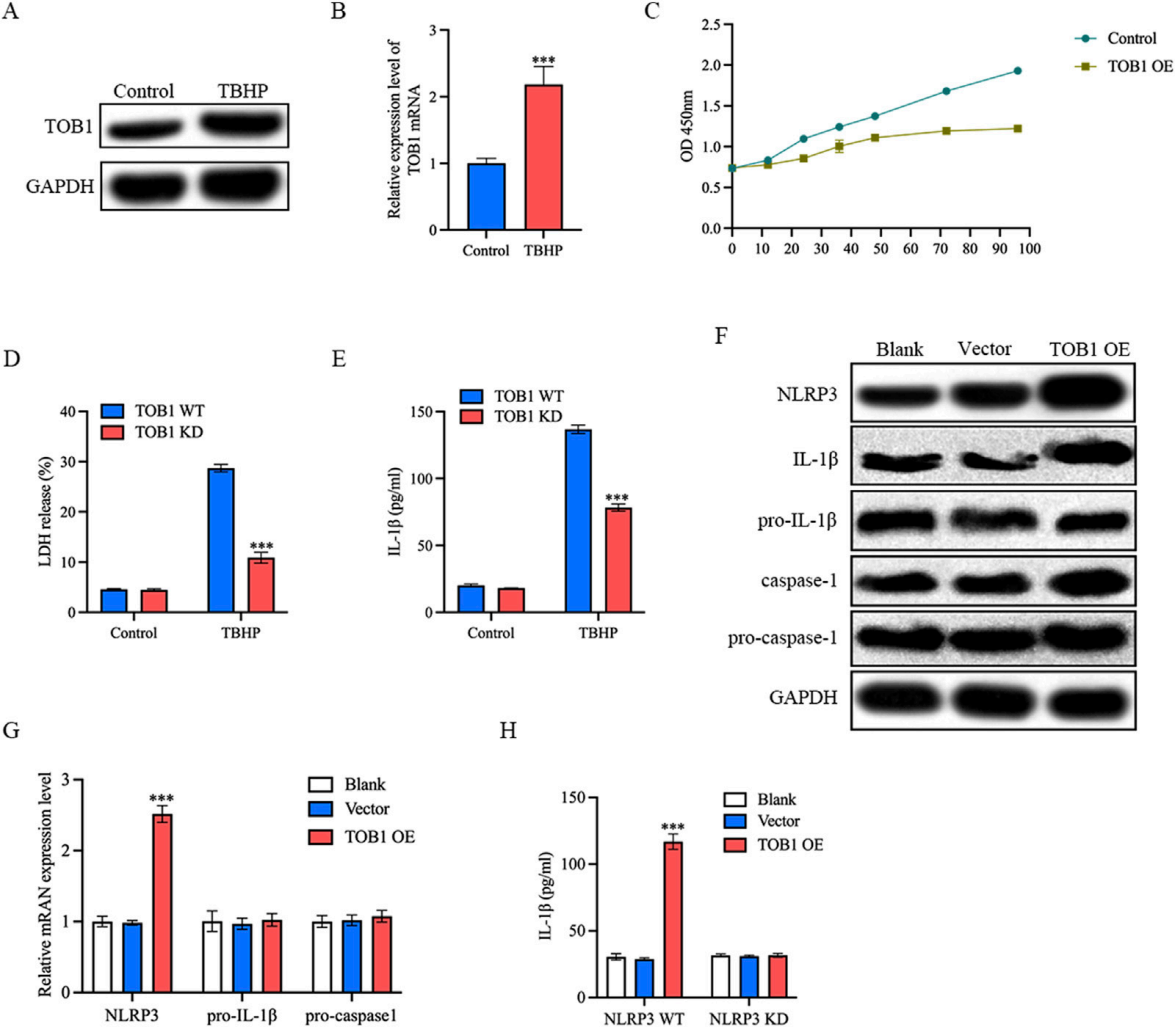
TOB1 overexpression activated NLRP3 inflammasome **(A and B)** NP cells were stimulated with TBHP. TOB1 protein and mRNA expression was assessed by Western blot and RT-qPCR. **(C)** NP cell proliferation ability was detected by CCK-8. **(D)** Pyroptosis was determined by LDH assay. **(E)** NP cells were transfected as indicated. IL-1β released was assessed by ELISA. **(F)** The Western blot assay for NLRP3, IL-1β cleavage and caspase-1 activation. **(G)** NP cells were transfected as indicated. NLRP3, pro- IL-1β and pro-caspase-1 mRNA were assessed by RT-qPCR. **(H)** NP cells were transfected as indicated. IL-1β released was assessed by ELISA.

Oxidative stress is one of the main driving mechanisms of IVD degeneration (Chen et al., 2024). Oxidative stress has been associated with inflammation in the IVD, cellular senescence, autophagy, and epigenetics of IVD cells (Chen et al., 2024). As shown in Figures 10A,B, TBHP increased TOB1 protein and mRNA expression level in comparison with control group. To assess the impact of TOB1 on NP cell proliferation, we performed a CCK-8 assay. Over six time points (12, 24, 36, 48, 72, and 96 h), our results indicated that the TOB1 overexpression significantly inhibited NP cell proliferation (Figure 10C). In this study, we also found that the absence of TOB1 markedly attenuated the release of LDH from NP cells (Figure 10D). IL-1β secretion following exposure to TBHP was significantly decreased in TOB1 knockdown NP cells as compared to wild-type NP cells (Figure 10E), suggesting that TOB1 had a regulatory effect on NP cells pyroptosis. Pyroptosis is a programmed inflammatory cell death that occurs when inflammasome is activated. To elucidate the effect of TOB1 in the activation of NLRP3 inflammasome, we examined IL-1β cleavage and caspase-1 activation in TOB1 overexpressed NP cells. The results showed that the activation of caspase-1 and the cleavage of IL-1β were significantly increased in TOB1 overexpressed NP cells (Figure 10F). Notably, TOB1 overexpression significantly increased NLRP3 protein and mRNA expression level, but had no effect on pro-IL-1β and procaspase-1 expression (Figures 10F,G). And NLRP3 knockdown markedly inhibited IL-1β secretion in TOB1 overexpression NP cells (Figure 10H). These results indicated that TOB1 can promote the expression of NLPR3 and activate the NLRP3 inflammasome, resulting in the pyroptosis of NP cells and the release of inflammatory cytokines.

## Discussion

IVD degeneration is a primary cause of low back pain, resulting in significant societal and financial burden (Conger et al., 2022). Low back pain is a prevalent reason for medical attention (Traeger et al., 2021). As the elderly population increases, there is an immediate need to clarify the origin and discover the optimal treatment for IVD degeneration. Up to now, IVD degeneration diagnosis primarily depends on symptoms and imaging, which makes early diagnosis and timely treatment difficult. Despite extensive research, the precise mechanisms of IVD degeneration remain obscure, and effective treatments are absent. Although some biomarkers have been detected in prior investigations, there has been no emphasis on the thorough exploration of immune cells and signature genes in IVD degeneration. Increasing evidence have demonstrated that immune cell infiltration acts as an important role in IVD degeneration (Wang et al., 2021). This study is the first time to reveal the relationship between TOB1 and immune cell infiltration during IVD degeneration.

TOB1 is a member of the TOB/BTG anti-proliferative (APRO) protein family, which regulates cellular quiescence and inhibits cell proliferation (Zhang et al., 2023). These proteins have been found in various cell types (Tzachanis and Boussiotis, 2009). TOB1 has been shown to inhibit cell growth and plays distinct regulatory roles in RNA processing in different types of cells, including neuronal cells, fibroblasts, and epithelial cells, based on compelling evidence (Matsuda et al., 2001; Jia and Meng, 2007; Perez et al., 2024). Juthika et al. study showed that TOB1 induced BAX expression and suppressed BCL-2 expression in gastric cancer cells (Kundu et al., 2012). In addition, TOB1 promoted caspase-3 activity, resulting in PARP cleavage and cellular apoptosis (Kundu et al., 2012). Research conducted by Xu et al. revealed that TOB1 plays a crucial part in infiltrating of T cells and involved in T cell functions (Xu et al., 2023). In this study, our results indicated that TOB1 has the ability to regulate immune response. Previously research demonstrated that IVD degeneration is characterized by infiltration of CD68^+^ macrophages, T cells (CD4^+^, CD8^+^), and neutrophils (Wang et al., 2021). scRNA-seq enables the identification of cell heterogeneity, allowing for a detailed analysis of biological structures and functions. This technique provides expression profiles for individual cells, facilitating the study of diverse cell populations and the classification of cell types. Understanding the phenotypes of immune cells within the IVD microenvironment is essential for unraveling the mechanisms behind IVD degeneration. This research revealed a significant increase in the infiltration of CD8^+^ T cells, activated NK cells, and neutrophils in degenerative NP tissues compared to control samples. A strong positive correlation was observed between TOB1 levels and the infiltration of these immune cells, indicating TOB1’s association with immune responses in IVD degeneration. Pyroptosis of NP cells leads to the release of inflammatory factors like IL-1β, which promotes immune cell infiltration and triggers an immune response. This immune response plays a crucial role in IVD degeneration, contributing to various pathological processes that result in fibrosis of NP tissue (Wang et al., 2020). Additionally, immune cells entering the IVD microenvironment release further inflammatory mediators, exacerbating damage and promoting the death of nucleus pulposus cells (Silva et al., 2019).

NLRP3 is a pattern recognition receptor that plays a crucial role in activating the innate immune system through the assembly of the NLRP3 inflammasome. This process involves the recruitment of the inflammasome complex by caspase-1, which is subsequently activated. Activated caspase-1 cleaves the IL-1β precursor protein, converting it into its biologically active form and promoting an immune response. In our research, we observed that TOB1 was highly expressed during IVD degeneration. TOB1 overexpression associated with the infiltration of CD8^+^ T cells, NK cell activated and neutrophils. TOB1 overexpression also activate NLRP3 inflammasome which results in IL-1β release and NP cell pyroptosis. This may be due to the overexpression of TOB1 in NP cells inducing the expression of NLRP3 and the activation of inflammasome. Combining the results of this study with the conclusions of previous studies, we hypothesized that a special type of immune microenvironment is generated during the process of IVD degeneration by release of IL-1β from NP cells that recruit CD8^+^ T cells, NK cell activated and neutrophils. TOB1 activates NLRP3 inflammasome to release IL-1β, which upregulates endothelial adhesion molecules to enhance neutrophil/CD8+ T cell recruitment. This aligns with high cytokine expression in pro-inflammatory macrophage subsets from single-cell data. This immune microenvironment, in turn, further promotes NP cell death and IVD pathological changes.

Our meticulous bioinformatic analysis provided a signature gene, namely, TOB1, which showed prominent value in diagnosis of IVD degeneration, and created opportunities for exploring therapeutic strategies. Besides, this study also explored the immune cell infiltration in IVD degeneration and their correlation with signature gene, which provided a new perspective for the role of immunity in IVD degeneration. Despite this, our study has limitations including small sample sizes from public datasets which may result in biased interpretations. However, the reliability of our analysis was confirmed by the results of *in vitro* experiments.

In conclusion, the present study identified a novel signature genes TOB1 and revealed that IVD degeneration samples immune infiltration landscape is markedly differed from healthy samples. In addition, according to this overall bioinformatic analysis, this study recognized critical regulatory pathways and immune infiltration characteristics of IVD degeneration. *In vitro* experiments, we identified TOB1 as a signature gene, which present good diagnostic value and may serve as a molecular target for treatment of IVD degeneration.

## Data Availability

The original contributions presented in the study are publicly available. This data can be found here: https://www.ncbi.nlm.nih.gov/geo/query/acc.cgi?acc=GSE56081 and https://www.ncbi.nlm.nih.gov/geo/query/acc.cgi?acc=GSE70362.
