# Supplementary material for: Identification of signature genes and relationship with immune cell infiltration in intervertebral disc degeneration
Source: Front Genet. 2025 Apr 9;16:1551124. doi: 10.3389/fgene.2025.1551124 (PMC12015982; doi:10.3389/fgene.2025.1551124)
Supplement: Supplementary file 4 [file Table4.DOCX]

**Table S4. Primer sequence for RT-qPCR**

| Gene | Forward Primer (5′-3′) | Reverse Primer (5′-3′) |
| --- | --- | --- |
| TOB1 | TCTGCTGCTGTAAGCCCTACCT | TTCATTTTGGTAGAGCCGAACTT |
| NLRP3 | GATCTTCGCTGCGATCAACAG | CGTGCATTATCTGAACCCCAC |
| Pro-IL-1β | ATGATGGCTTATTACAGTGGCAA | GTCGGAGATTCGTAGCTGGA |
| Pro-caspase-1 | TTTCCGCAAGGTTCGATTTTCA | GGCATCTGCGCTCTACCATC |
| GAPDH | ACAACTTTGGTATCGTGGAAGG | GCCATCACGCCACAGTTTC |
